# *Saccharomyces cerevisiae* Cell Wall-Based Adsorbent Reduces Aflatoxin B1 Absorption in Rats

**DOI:** 10.3390/toxins13030209

**Published:** 2021-03-13

**Authors:** Alexandros Yiannikouris, Juha Apajalahti, Osmo Siikanen, Gerald Patrick Dillon, Colm Anthony Moran

**Affiliations:** 1 Chemistry and Toxicology Division, Center for Animal Nutrigenomic and Applied Animal Nutrition, Alltech Inc., 3031, Nicholasville, KY 40356, USA; 2 Alimetrics Ltd., Koskelontie 19B, 02920 Espoo, Finland; j.apajalahti@alimetrics.com (J.A.); osmo.siikanen@alimetrics.com (O.S.); 3 Alltech Ireland, Sarney, Summerhill Road, A86 X006 Dunboyne, Ireland; gdillon@alltech.com; 4 Alltech SARL (France), Rue Charles Amand, 14500 Vire, France; cmoran@alltech.com

**Keywords:** mycotoxins, aflatoxin B1, yeast cell wall, binder, animal health, absorption, adsorption

## Abstract

Mycotoxins are naturally occurring toxins that can affect livestock health and performance upon consumption of contaminated feedstuffs. To mitigate the negative effects of mycotoxins, sequestering agents, adsorbents, or binders can be included to feed to interact with toxins, aiding their passage through the gastrointestinal tract (GI) and reducing their bioavailability. The parietal cell wall components of *Saccharomyces cerevisiae* have been found to interact in vitro with mycotoxins, such as, but not limited to, aflatoxin B1 (AFB1), and to improve animal performance when added to contaminated diets in vivo. The present study aimed to examine the pharmacokinetics of the absorption of radiolabeled AFB1 in rats in the presence of a yeast cell wall-based adsorbent (YCW) compared with that in the presence of the clay-based binder hydrated sodium calcium aluminosilicate (HSCAS). The results of the initial pharmacokinetic analysis showed that the absorption process across the GI tract was relatively slow, occurring over a matter of hours rather than minutes. The inclusion of mycotoxin binders increased the recovery of radiolabeled AFB1 in the small intestine, cecum, and colon at 5 and 10 h, revealing that they prevented AFB1 absorption compared with a control diet. Additionally, the accumulation of radiolabeled AFB1 was more significant in the blood plasma, kidney, and liver of animals fed the control diet, again showing the ability of the binders to reduce the assimilation of AFB1 into the body. The results showed the potential of YCW in reducing the absorption of AFB1 in vivo, and in protecting against the damaging effects of AFB1 contamination.

## 1. Introduction

Mycotoxins are major natural contaminants present in food and feed materials, such as grains or forages [1,2]. The spores of mycotoxin-producing fungi are ubiquitous in the environment, hence, they inevitably contaminate grains and other plant-based feed materials [3]. Under high humidity, moderate temperature, and aerobic conditions, spores can germinate and grow. Under specific biotic and abiotic stress conditions, some can release mycotoxins as secondary metabolites directly to plants or stored ingredients [4]. Moreover, environmental challenges, such as meteorological events, the plant health status, and suboptimal storage conditions of feed materials, can lead to increased access to nutritious substrates, permitting fungal growth and the promotion of certain mycotoxin-producing fungal species. This can result in the increased production and release of mycotoxin varieties and concentrations [5]. Mycotoxins, especially aflatoxins (comprising aflatoxin B1 (AFB1), -B2, -G1, and -G2) and, in particular, AFB1 (a metabolite of some *Aspergillus* fungal species), are potent hepatotoxic and hepatocarcinogenic toxins. These were discovered following the outbreak of Turkey X disease in England in 1960, which launched an extensive array of scientific investigations into the field of mycotoxins [6]. Aflatoxins are poorly degraded in the monogastric and ruminal digestive systems after ingestion and are rapidly absorbed (up to 90%) in the proximal part of the digestive tract, notably the duodenum. Following absorption, plasmatic proteins, such as albumin, can carry aflatoxins and transport them to the liver [7]. The metabolization of AFB1 has been well characterized; it involves a two-phase metabolization process [8]: (i) a phase 1 bioactivation by enzymatic epoxidation (microsomal cytochrome P450 (CYP)) of AFB1 into 8,9-epoxy-aflatoxin B1 (8,9-epoxy-AFB1), which can form adducts with nucleic acids in DNA or undergo further hydrolysis into epoxy-AFB1-dihydrodiol, or a reversible enzymatic reduction (AFB1-reductase NADPH-dependent) into aflatoxicol; (ii) a phase 1 biodetoxification by oxidation (microsomal CYP) of AFB1 into less-toxic metabolites, such as aflatoxin M1, -P1, -Q1, and -H1; and (iii) a phase 2 metabolization, a critical pathway to detoxification, via enzymatic conjugation (glutathione S-transferase) to glutathione of 8,9-epoxy-AFB1 or of the oxidized forms (UDP-glucuronosyltransferases, sulfotransferases) to glucuronic acid or sulphonic acid for further elimination via urine. As another route of elimination, fat-soluble toxins can be excreted via milk, where AFM1 is the major milk metabolite of aflatoxin B1 [9].

AFB1 can impair animal performance following a subacute or chronic exposure and known to cause mortality in acute aflatoxicosis cases, with a large variability observed between animal species. The LD50 values vary from 0.3 mg/kg body weight (BW) in ducklings to 9.0 mg/kg BW in mice and up to 17.9 mg/kg BW in female rats [10,11]. Responses to chronic exposure of AFB1 can fluctuate widely between and within animal species. Much depends on animal gut health integrity and the presence of stressors, such as bacterial pathogens, and the animal species-dependent microsomal biotransformation activity. The latter activity can either promote biodetoxification or bioactivation, ultimately affecting the final genotoxicity, immunotoxicity, and further carcinogenicity of the toxin. AFB1 has also been found to trigger nutrient depletion, such as vitamins A, D, and E, and indirectly impairs growth [7], disrupts cell function, and exerts immunotoxicity [12]. AFB1 has been classified as a group 1 carcinogen by the International Agency for Research on Cancer (IARC), and AFM1 as group 2B. In animals, the permissible levels of AFB1 are tightly regulated as undesirable substances in feedstuffs [13,14] based on the feed ingredient type, animal species, and age group.

To reduce mycotoxins’ effect on animal performance and minimize AFB1 exposure in animals, sequestering, adsorbent, or binding agents are often added to AFB1-contaminated feedstuffs as a mitigation strategy. This dietary strategy is implemented to overcome the high resistance of AFB1 to many physical and chemical means of decontamination, in addition to integrated strategies for reducing mold contamination and mycotoxin occurrence in feed production [15]. Binders (i.e., adsorbents and sequestrants) include activated carbon, inorganic mineral clays, organically modified clays, and yeast cell wall preparations [15,16,17,18]. An effective adsorbent can bind one or several mycotoxins in an animal’s digestive tract and keep the toxin bound during passage through the animal gastrointestinal tract. Thus, the quantity of bioavailable mycotoxin reaching an animal tissues or organs, and concomitantly impairing animal health, is reduced [19]. Several mycotoxin binders, particularly a yeast cell wall-based adsorbent (YCW), have shown the ability to successfully adsorb different groups of mycotoxins, as demonstrated by in silico computational mechanistic studies characterizing their chemical interactions [20,21,22], in an in vitro study characterizing their kinetics in reaction media [23], and in ex vivo studies using intestinal tissues [24,25]. Multiple in vivo studies of various animal species have shown the mitigation capabilities of binders following the administration of synthetic or natural mycotoxins via diet, whereas the binder’s efficacy has been appraised based on an observed improvement in animal performance [26,27,28]. Other animal studies have primarily focused on indirect, often non-specific/shared biomarkers of exposure as an outcome in the evaluation of mitigation strategies, such as measuring changes in intestinal health using histopathological assessment [29], modification of blood chemistry [30,31,32,33], changes in immunological titers [34,35,36,37,38], changes in microbiota [32,39], genomic and antioxidant markers [40,41,42,43], and changes in organ morphology [44,45]. However, only a few in vivo studies have measured toxin partitioning in the animal body and revealed the pharmacokinetics of toxin accumulation in different tissues and digesta [46,47,48].

Therefore, in the present study, we aimed to assess the efficiency of YCW as a binder for AFB1 compared with that of hydrated sodium calcium aluminosilicate (HSCAS), which has been previously shown to have high affinity specifically for AFB1 [18,49]. For this purpose, after evaluating the characteristics of both YCW and HSCAS adsorbents toward AFB1 in vitro, we assessed the effect of YCW on AFB1 absorption in vivo in a rat model. Prior to the main animal study, a preliminary study was conducted to reveal the kinetics of AFB1 absorption with a specific diet and to optimize the sampling time points that would be further used. In the main study, the distribution of radiolabeled AFB1 in digesta (the stomach, small intestine, cecum, and colon) and systemic tissues (the plasma, liver, and kidney) was measured in the presence and absence of a commercial source of YCW, Mycosorb^®^.

## 2. Results

### 2.1. In Vitro Preliminary Study of the Adsorption Capacity of the Tested Adsorbents toward AFB1

**Figure 1:**
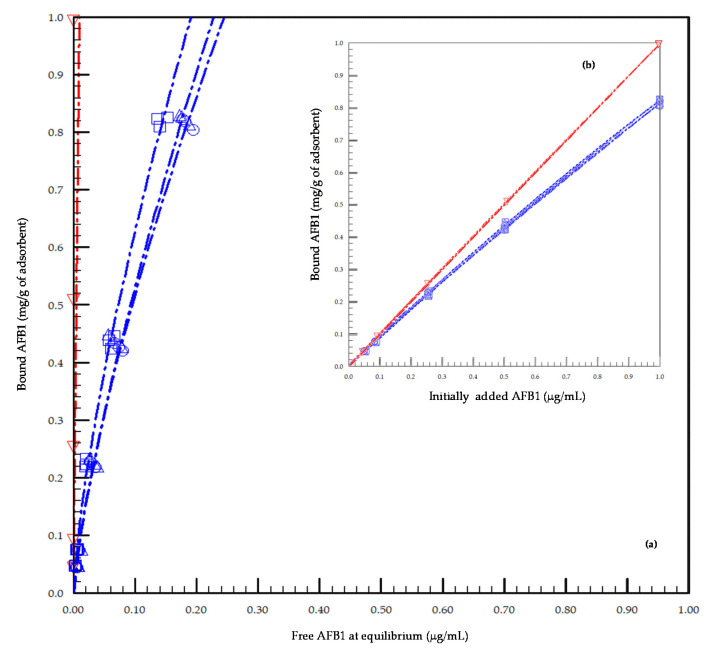
Concentrations of bound vs. free aflatoxin B1 (AFB1) evaluated at pH 3.0 using three independent batches of yeast cell wall-based adsorbent (YCW) or one hydrated sodium calcium aluminosilicate (HSCAS). (**a**) The concentration of free AFB1 at equilibrium is expressed as µg/mL, and the corresponding bound concentration of AFB1 is expressed as mg/g for each adsorbent used: YCW (open blue squares, circles, triangles) and HSCAS (red triangles) at pH 3.0; ((**b**) subfigure window) the concentration of the initially added AFB1 is expressed as µg/mL, and the corresponding bound concentration of AFB1 at equilibrium is expressed as mg/g for each adsorbent used: YCW (open blue squares, circles, triangles) and HSCAS (red triangles) at pH 3.0. All replicate values (three replicates per concentration tested for each individual product) are displayed in the graphic. Adsorption curves were fitted using the Freundlich equation.

The percentage of AFB1 bound on an individual basis to each tested concentration (Table 1) of each adsorbent tested ranged from 81% to 94% for YCW and was 100% for HSCAS. The average adsorbed percentage for YCW was approximately 89% of the AFB1 present in the medium when tested at pH 3.0, which differed significantly from that for HSCAS (*p* < 0.0001) with 100% AFB1 adsorption. The coefficients of variation obtained for YCW were <5% and 0.01% for HSCAS. Regression analyses were performed on data for the three batches of YCW and one of HSCAS using three models recommended by FEFANA to test the adsorption properties of the adsorbents [50], however, using sub-ppm levels of AFB1 ranging from 0.05 to 1.00 ng/mL (Figure 1). All models fitted the data points with a regression coefficient above 0.9760. Hill’s model with *n* sites being the best fitting model for all the YCW-tested materials (0.9853), however, the overall models were difficult to differentiate for the tested concentrations. Using Freundlich equation, we determined that the average adsorption capacity *K*_F_ values of YCW and HSCAS were 3.06 and 1.03, respectively. Using Hill’s model, where the cooperativity of the interaction can be evaluated, there was little difference between adsorbents, as the *n* value averaged across sorbents at 0.94 ± 0.25, showing a linear behavior of the model for the tested concentration bracket.

### 2.2. In Vivo Experiment on the Total Recovery of AFB1 in Rat Fed AFB1-Contaminated Diet

The preliminary study results showed that ^3^H-AFB1 levels increased after 2–3 h in all samples, except for those in the duodenal/jejunal samples and, to a lesser extent, in the ileal digesta, where the levels were constant or reached a plateau. In most of the tissues analyzed, the peak absorption level was not reached even at the final time point of 7 h. Based on the preliminary study results, the time points of 5 and 10 h were selected for the final study. Before 5 h, the accumulation of AFB1 in systemic tissues (the plasma, liver, and kidney) was expected to be low. Therefore, the probability of achieving significant effects was estimated to be poor. A 10-h collection point was chosen to obtain information on the distribution of AFB1 between digesta and tissues at a later time point. A considerable portion of the tested toxin was anticipated to be excreted via urine and feces. As a separate analysis of duodenal/jejunal and ileal digesta samples did not yield any additional information, they were combined in the final study as ‘small intestine’. We decided to enlarge the study’s scope to encompass the stomach and cecum samples to expand the recovery profile of AFB1.

**Figure 2:**
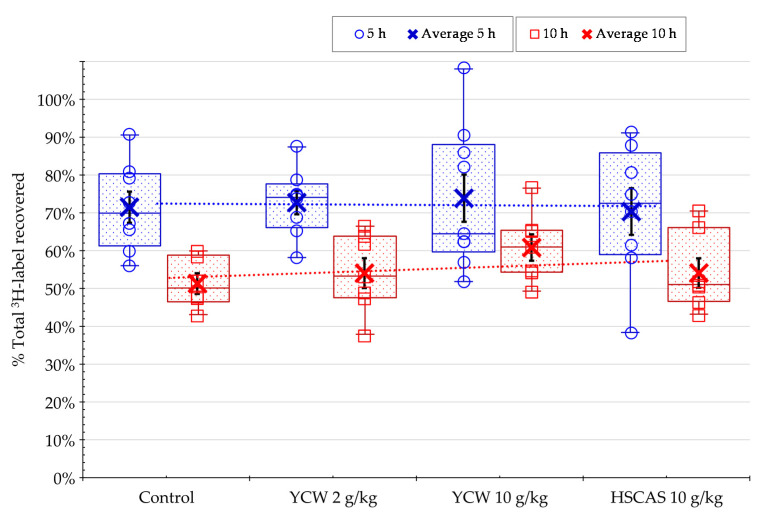
Total recovery of the ^3^H-label from ^3^H-aflatoxin B1 (^3^H-AFB1) expressed as the percentage of the initial dose administered in all samples analyzed after the oral administration of AFB1-contaminated diet to rats in the presence or absence of yeast cell wall-based adsorbent (YCW) or hydrated sodium calcium aluminosilicate (HSCAS) at different concentrations. All replicate (open circles/squares) and average values (cross) are displayed in the graphic: (**1**) Box and whisker chart, as well as median (horizontal line), average (cross) and quartiles calculations (box); and (**2**) the regression curve of the average values shows the magnitude of the recovery. Bars (in black) in boxes correspond to the standard errors of the mean of the replicate rats. The study was performed initially on *n* = 64 rats, or 16 rats per treatment. At 5 h (in blue), *n* = 9 rats for the 10 g/kg YCW treatment and *n* = 8 for the rest of the treatments were collected for analysis; At 10 h (in red), the reminder rats (4 rats were excluded due to morbidity/mortality issues before the start of the main experimental study period) per treatments were collected for analysis, *n* = 6 in the control group and *n* = 7 in each of the adsorbent treated groups.

In the main study, at 5 and 10 h post-feeding, the rats were sacrificed, and the radioactivity measured in the different tissues sampled. The distribution of the radiolabel signal in the digesta of the various sections of the gastrointestinal tract, plasma, liver, and kidney showed an average recovery at 5 and 10 h of 72% and 55%, respectively, of the total ^3^H-AFB1 administered via diet (Figure 2).

### 2.3. Evaluation of the Absorption Kinetics of AFB1 in Rat Fed AFB1-Contaminated Diet

**Figure 3:**
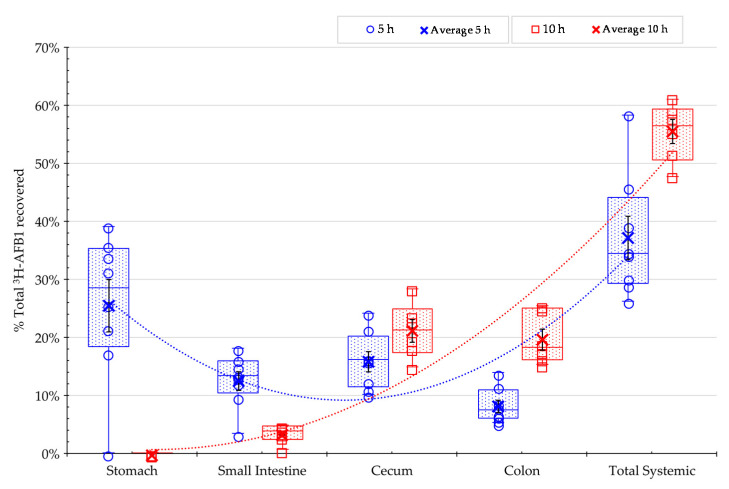
Distribution of the ^3^H-label from ^3^H-aflatoxin B1 (^3^H-AFB1) in rats at 5 (in blue) and 10 h (in red) after administration of the control treatment. Figures indicate the percentages of the total radiolabeled AFB1 recovered from digesta in different intestinal compartments and the total systemic radiolabeled AFB1 (the sum of radioactivity in the plasma, liver, and kidney). All replicate (open circles) and average values (cross) are displayed in the graphic: (**1**) Box and whisker chart, as well as median (horizontal line), average (cross), and quartiles calculations (box); and (**2**) the regression curve of the average values shows the magnitude of the recovery. Bars (in black) in boxes correspond to standard errors of the mean of the replicate rats. Control treatment initially comprised 16 rats. The integrality of each gastrointestinal compartiment was collected for: *n* = 8 rats at 5 h; the reminder *n* = 6 rats at 10 h (two rats were excluded from this analysis due to morbidity/mortality issues before the start of the main experimental study period) for analysis.

The kinetics of AFB1 absorption was assessed by measuring toxin distribution in selected tissues and intestinal digesta. As shown in Figure 3, ^3^H-AFB1 was found in high abundance in the stomach (~26%) and small intestine (~13%) after 5 h post-feeding but was observed in low abundance of ~4% at 10 h post-feeding. In contrast, the amount of ^3^H-AFB1 in the cecum and colon increased at 10 h, even though significant absorption to tissues had occurred (Figure 3).

This finding reflected the overall evolution of the ^3^H-AFB1 digesta transit from the proximal to distal compartments of the gastrointestinal tract. At the 5 h time point, 35% of the recovered label was found in the systemic tissues comprising the plasma, liver, and kidney, whereas the proportion increased to 55% in the same tissues at 10 h after AFB1 feeding. The results indicated that the bulk of aflatoxin present was absorbed in the gastrointestinal tract.

### 2.4. Effect of Mycotoxin Adsorbents on AFB1 Retention in the Gastrointestinal Tract

**Figure 4:**
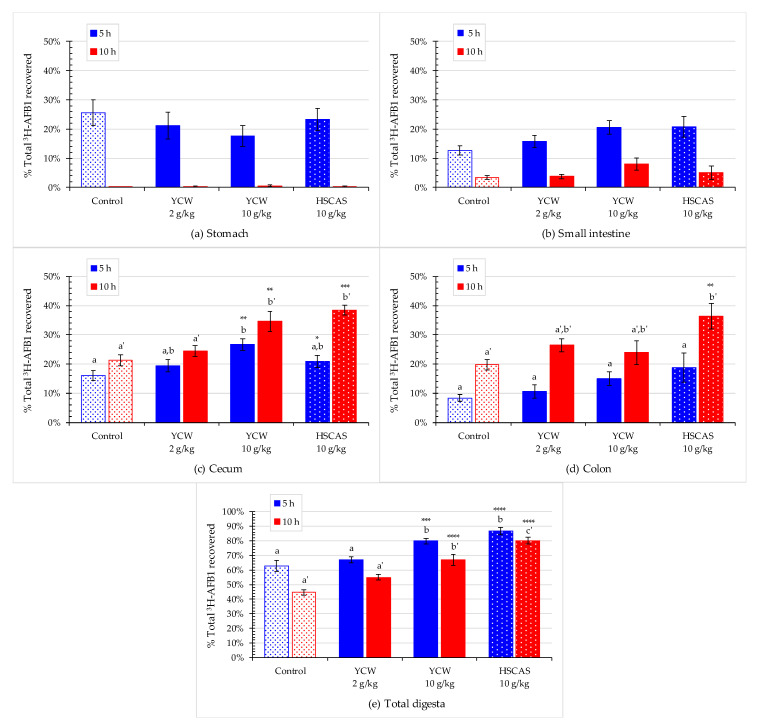
The effect of mycotoxin binders on the residual level of the ^3^H-label from ^3^H-aflatoxin B1 (^3^H-AFB1) in digesta at 5 (in blue) and 10 h (in red) after toxin administration with or without the addition of yeast cell wall-based adsorbent (YCW) at two concentrations or hydrated sodium calcium aluminosilicate (HSCAS). Panels (**a**–**e**) show the percentage of recovered ^3^H-AFB1 found in the (**a**) stomach, (**b**) small intestine, (**c**) cecum, (**d**) colon, and (**e**) total digesta. Bars in the columns correspond to standard errors of the mean of the replicate rats. The significant difference between the control and amended feeds are indicated by asterisks as follows: * 0.01 ≤ *p* value < 0.05; ** 0.001 ≤ *p* value < 0.01; *** 0.001 ≤ *p* value < 0.001; **** *p* value < 0.0001 using Dunnett’s post-hoc test. In addition, pairwise comparisons were tested by Tukey’s post-hoc test. Different letters indicate significant difference between treatments within a sampling time point. Study was performed initially on *n* = 64 rats or 16 rats per treatment. At 5 h, *n* = 9 rats for the 10 g/kg YCW treatment and *n* = 8 for the rest of the treatments were collected for analysis; At 10 h, the reminder rats (four rats were excluded due to morbidity/mortality issues before the start of the main experimental study period) per treatments were collected for analysis, *n* = 6 in the control group and *n* = 7 in each of the adsorbent treated groups. Integrality of each digestive compartiment and systemic tissue was collected for each rat.

Evaluation of the binder strategy’s effect involved comparing the adsorbents with a control diet supplemented only with AFB1. Figure 4a–d show the sequential evolution of the recovery rate of ^3^H-AFB1 in the digesta collected from the stomach, small intestine, cecum, and colon. At 5 h, more than 20% of the recovered radiolabeled AFB1 was found in the stomach (Figure 4a). No differences in recovery were observed between the respective dietary treatments, suggesting that the stomach was not a significant place of AFB1 absorption. Hence, any portion of toxin present in this compartment would remain in the digesta. At the 10 h timepoint, the stomach compartment was empty, and no detectable levels of ^3^H-AFB1 were found in the samples from any treatment.

In the small intestine, the apparent recovery rate of ^3^H-AFB1 tended to numerically increase with the addition of an adsorbent, at 5 h, with an increase from ~12% in the control to 15% in rats fed 2 g/kg of YCW, and to ~20% in rats fed 10 g/kg of YCW or HSCAS (Figure 4b). A similar trend was observed at 10 h post-feeding, but the level of AFB1 recovered fluctuated between ~3% and ~8%, respectively, for the control and 10 g/kg YCW groups. The effects were not significant at the risk levels used in the Dunnett’s and Tukey’s post-hoc tests. However, the multiple linear regression (MLR) model showed a significant dose-dependent effect using YCW at both time points (Table 2 and Table 3).

The radioactive recoveries in the cecal digesta showed a similar effect to those observed in the small intestine. In contrast, higher recoveries were obtained with the diets containing the mycotoxin binders compared with those obtained with the control diet, showing increases at 5 h post-feeding from ~16% in the control group up to ~28% in the 10 g/kg YCW group and from ~21% in the control group up to 39% in the HSCAS group. However, the results showed higher significance than those in the small intestine (Figure 4c, Table 2 and Table 3). The effect of HSCAS and YCW supplementation at 10 g/kg was almost identical in both the small intestine and cecum at the 5-h time point. Conversely to the small intestine and as described previously, toxin concentration in the cecum was higher at the 10 h timepoint. This indicated that at 10 h post-feeding, the small intestine started to empty, whereas the digesta content of the cecum and colon remained high. In the cecum, the increase in the AFB1 content was significantly higher with YCW at 5 h (*p* < 0.01) than with HSCAS (*p* < 0.05), which revealed a potentially higher adsorption affinity for YCW. At 10 h, the AFB1 content was significantly higher with HSCAS treatment (*p* < 0.001) than YCW treatment (*p* < 0.01), which showed a potentially higher capacity of adsorption for HSCAS.

In the colon, toxin retention tended to increase with adsorbent use, but this increase was not significant. HSCAS at 10 h showed a significant increase in toxin retainment compared with the control, but YCW did not (Figure 4d). There was no significant difference in toxin retainment at 10 h post-feeding in the colon between the YCW and control groups.

The total levels of recovered ^3^H-AFB1 in the different digesta of the gastrointestinal tract highlighted a dose-dependent toxin-binding effect of YCW and HSCAS. Treatment with the binders at 10 g/kg led to a significant increase in AFB1 detected in the total digesta (*p* < 0.001). The overall effect of both products tested was highly significant at both time points (Figure 4e, Table 2 and Table 3).

### 2.5. Effect of Mycotoxin Binders on AFB1 Absorption into Animal Tissues

**Figure 5:**
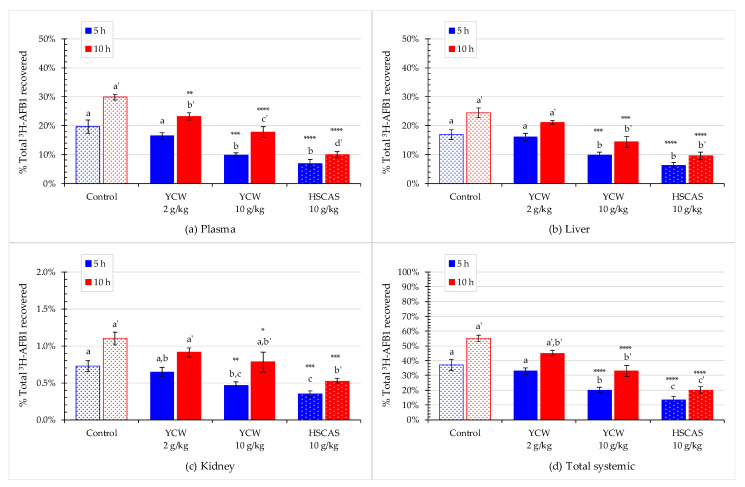
The effect of mycotoxin binders on the residual level of the ^3^H-label from ^3^H-aflatoxin B1 (^3^H-AFB1) in digesta at 5 (in blue) and 10 h (in red) after toxin administration with or without the addition of yeast cell wall-based adsorbent (YCW) at two concentrations or hydrated sodium calcium aluminosilicate (HSCAS). Panels (**a**–**d**) show the percentage of recovered radioactivity found in the (**a**) plasma, (**b**) liver, (**c**) kidney, and (**d**) total systemic. Bars in columns correspond to standard errors of the mean of the replicate rats. Significant differences between the control and amended feeds are indicated by asterisks as follows: * 0.01 ≤ *p* < 0.05; ** 0.001 ≤ *p* <0.01; *** 0.001 ≤ *p* < 0.001; **** *p* < 0.0001 as analyzed using Dunnett’s post-hoc test. In addition, pairwise comparison was tested by Tukey’s post-hoc test; different letters indicate significant difference between treatments within a sampling time point. Study was performed on initially *n* = 64 rats or 16 rats per treatment. At 5 h, *n* = 9 rats for the 10 g/kg YCW treatment and *n* = 8 for the rest of the treatments were collected for analysis; At 10 h, the reminder rats (four rats were excluded due to morbidity/mortality issues before the start of the main experimental study period) per treatments were collected for analysis, *n* = 6 in the control group and *n* = 7 in each of the adsorbent treated groups. Integrality of each digestive compartiment and systemic tissue was collected for each rat.

In the present study, AFB1 absorption was analyzed in a fixed volume of blood and then calculated to estimate the aflatoxin absorption in the entire volume of blood in rats [51]. Analysis of blood plasma samples showed that the diets containing a mycotoxin binder significantly reduced the concentration of recovered ^3^H-AFB1 in a dose-dependent manner (Figure 5a). At the 5-h time point, the diets containing YCW and HSCAS at 10 g/kg reduced the toxin concentration by ~50% (*p* < 0.001) and ~65% (*p* < 0.0001), respectively, compared with the control diet. These two treatments did not differ significantly from each other but differed from the control. At the 10-h timepoint, 30% of the labeled aflatoxin was found in the rats’ plasma fed the control diet. The diets containing 2.0 g/kg (*p* < 0.01) and 10 g/kg (*p* < 0.0001) YCW as well as the diet containing HSCAS (*p* < 0001) showed a respective reduction in plasma AFB1 of ~20%, ~40%, and ~65%. At this time point, the responses of all four feeds differed significantly from each other, and the MLR model (Table 3) for the YCW dose responses were also highly significant (*p* < 0.0001).

YCW and HSCAS at 10 g/kg significantly reduced the toxin concentration in the liver (*p* < 0.0001) by ~40% and ~60%, respectively, at both time points (Figure 5b, Table 2 and Table 3). There was no significant reduction in the toxin concentration in the 2.0 g/kg YCW group than in the control group.

At the 5- and 10-h timepoints, only 0.7% and 1% of ^3^H-AFB1 were found in the control rats’ kidneys. Even though the total radioactivity in the kidneys represented only a small proportion of the total radioactivity, the two tested products’ effects were similar to those observed in the plasma and liver, with a decrease in the accumulated levels. Again, HSCAS (*p* < 0.001) and YCW (*p* < 0.05) significantly reduced the level of radiolabeled aflatoxin at both time points (Figure 5c). However, when administered at 10 g/kg, YCW and HSCAS exhibited no significant differences from one another at any post-feeding times.

Overall, both adsorbents significantly reduced the total systemic accumulation of AFB1 from ~47% in the control down to ~20% and ~15% after 5 h of exposure and from ~55% down to ~30 and ~20% after 10 h of exposure a following dietary treatments with YCW and HSCAS, respectively (Figure 5d).

**Figure 6:**
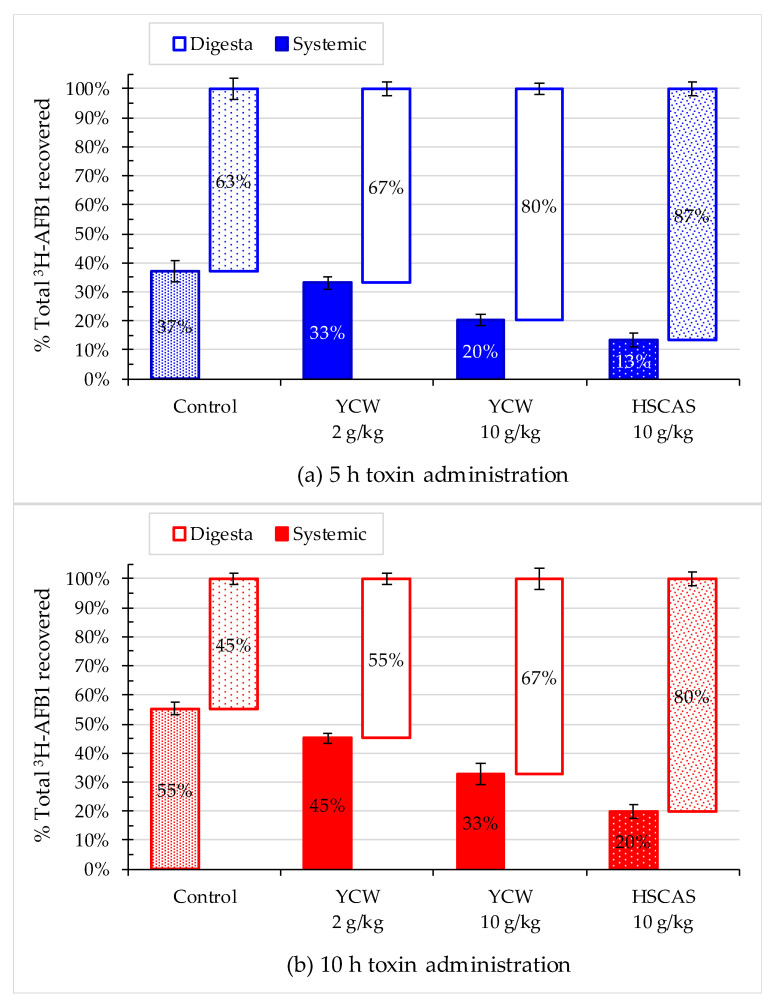
Distribution of the recovered the ^3^H-label from ^3^H-aflatoxin B1 (^3^H-AFB1) in rat tissues (systemic) and intestinal content (digesta) at (**a**) 5 h (blue) and (**b**) 10 h (red) after the toxin administration with or without the addition of yeast cell wall-based adsorbent (YCW) at two concentrations or hydrated sodium calcium aluminosilicate (HSCAS). Error bars indicate standard errors of the mean. This study was performed initially on *n* = 64 rats, or 16 rats per treatment. At 5 h, *n* = 9 rats for the 10 g/kg YCW treatment and *n* = 8 for the rest of the treatments were collected for analysis; At 10 h, the reminder rats (four rats were excluded due to morbidity/mortality issues before the start of the main experimental study period) per treatments were collected for analysis, *n* = 6 in the control group and *n* = 7 in each of the adsorbent treated groups. Integrality of each digestive compartiment and systemic tissue was collected for each rat.

When both digesta and systemic accumulation were evaluated in combination at the 5-h timepoint, ~60% and ~40% of the labeled aflatoxin were found respectively in the intestinal digesta and systemic samples of the animals fed the diet containing no mycotoxin binders (Figure 6). The two mycotoxin adsorbents significantly changed this distribution, with 80% of AFB1 recovered in digesta and only less than 20% in the tissue samples when HSCAS was introduced in the diet. Similarly, YCW at 10 g/kg reduced the proportion of absorbed AFB1 from 40% to 20%. At 10 h post-feeding, as high as 55% of AFB1 was recovered in the animals’ tissues fed the control diet. HSCAS also reduced the level of absorbed aflatoxin to 20% at the 10-h time point. YCW also significantly reduced the toxin absorption by 40%, thereby exerting a protective effect.

**Figure 7:**
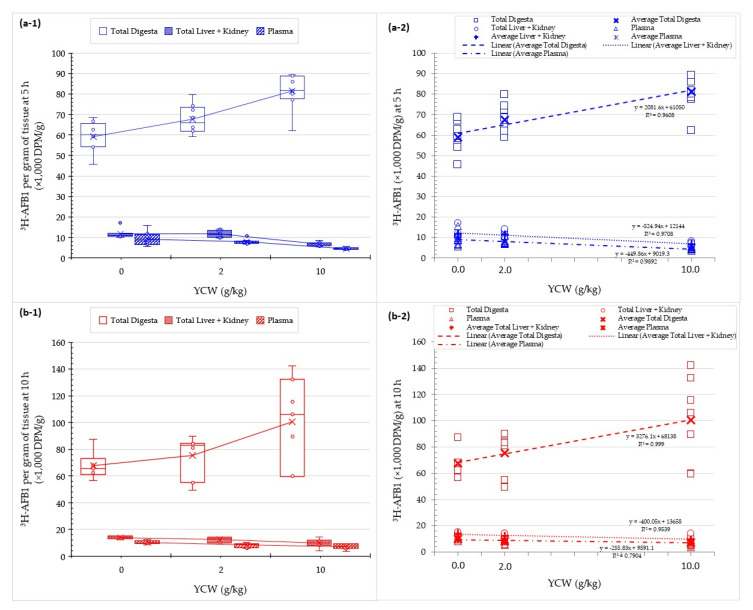
Dose response evaluation measured from the disintegration per minute of the ^3^H-label from ^3^H-aflatoxin B1 (^3^H-AFB1) normalized per gram of digesta or tissue collected (×1000 DPM/g) or per milliliter of plasma (×1000 DPM/mL) in rat at (**a-1**,**a-2**) 5 h (blue) and (**b-1**,**b-2**) 10 h (red) after the toxin administration with 0, 2, and 10 g/kg dose of yeast cell wall-based adsorbent (YCW). All data points measured are reported on: (**1**) Box and wiskers chart, as well as median (horizontal line), average (cross), and quartile calculations (box); and (**2**) the regression curve on the average values evaluating the direction and magnitude of the effect relative to the YCW dose. This study was performed initially on 16 rats per treatment. At 5 h, *n* = 9 rats for the 10 g/kg YCW treatment and *n* = 8 for the rest of the treatments were collected for analysis; at 10 h, the reminder rats (three rats were excluded from this analysis due to morbidity/mortality issues before the start of the main experimental study period) per treatments were collected for analysis, *n* = 6 in the control group and *n* = 7 in each of the YCW treated groups. Integrality of each digestive compartiment and systemic tissue was collected for each rat. All replicate (open circles/squares) and average values (cross) are displayed in the graphic.

When evaluating the effect of the 0, 2 and 10 g/kg dose response on YCW (Figure 7), we accounted for a linear increase in the ^3^H-AFB1 label in the digesta content and conversely a decrease of the label in the systemic tissue investigated. This representation confirmed the statistical results obtained with the MLR model, establishing a significant dose-dependent effect using YCW (Table 2 and Table 3).

## 3. Discussion

This study’s primary aim was to investigate the digestive and systemic distribution of AFB1 in the rat, in order to elucidate the bioavailability and the dispersal pattern of this mycotoxin. Based on a literature search, this is the first report describing the pharmacokinetics of AFB1 in different digestive compartments and organs. Several advantages were apparent through the application of tritium labelled AFB1 in this study. It allowed to map the overall aflatoxin distribution (including AFB1 and any metabolites thereof) without the need to develop complex analytical methodologies or account for subsequent recovery, separation, and detection variables. However, using this strategy, limitations arose from our inability to discriminate those species and define different AFB1 metabolite profiles within the animal compartment studied herein and how they could be influenced by the other dietary treatments evaluated.

In this study, we also assessed the efficiency of YCW as a binder for AFB1 compared to that of HSCAS. The in vitro evaluation of the adsorption properties of three batches of YCW and HSCAS, tested at pH 3.0 and 37 °C for 90 min, highlighted a very high interaction affinity of above 89% for YCW and 100% for HSCAS at the tested concentrations. This in vitro experiment differed from previous experimental methods, as it focused on field-levels of AFB1 concentrations in the sub-parts per million range. We confirmed the capacity of both materials to interact with AFB1 effectively, and that the affinity of interaction in the domain of definition of the tested concentration was almost linear, as defined by the slope of the curve using the Freundlich model, the model previously identified as most suited for comparing adsorbents of different nature [24,25]. This model generally defines adsorption events occurring on heterogeneous surfaces, making it more appropriate for a study of both YCW and HSCAS than the Langmuir equation, which is intended for gas-solid interaction [52]. Hill’s equation with *n* sites has been defined as a suitable equation to characterize cooperative phenomena that considers the dynamic changes influencing the number of binding sites on an adsorbent, which is particularly suitable for the study of YCW, as shown previously for different types of mycotoxins [25,53]. In the present study, little difference was observed among the various models owing to the linear behavior of the relationship between free and bound AFB1 at equilibrium, for the tested concentrations.

In the current animal experiment, the observation that a smaller proportion of ^3^H-AFB1 was recovered after 10 h suggested a significant portion of the label was lost. This deficit may be due to the biotransformation of the AFB1 molecule through microsomal activities, and the consequent loss of tritium, which could occur during the epoxidation of AFB1 into 8,9-epoxy-AFB1 (representing a loss of two hydrogens out of 14 per one AFB1 molecule, or a 15% tritium deficit) or biodetoxification following demethylation of AFB1 into AFP1 (three hydrogens lost or a 21% tritium deficit). Unfortunately, the rate of AFB1 biotransformation into those metabolites has not been adequately described in the literature, but it is known to vary according to the microsomal CYP polymorphism, with dominant bioactivation from CYP1A2 found in rodents and studied in human liver microsomes or genetically-modified yeast models [54,55]. The decrease in the recovery of radiolabeled AFB1 could be due to its excretion from the body via the urine or feces or the further partitioning into other tissues, e.g., muscle tissues, which were not analyzed in this study. The total radioactivity recovery rate was not affected by the rats’ diets, treatment type, or adsorbent concentration, as shown in Figure 2.

The kinetics of AFB1 absorption was assessed by measuring toxin distribution in selected tissues and intestinal digesta. The results showed a logical evolution of the ^3^H-AFB1 digesta transit from the proximal to the distal parts of the gastrointestinal tract. Surprisingly, the absorption process was relatively slow compared with protein digestion and absorption, which generally occur within 2 h in the gut’s proximal region. A previous study has shown that, when perfused, AFB1 tends to be absorbed in the duodenum. However, when investigated in vitro, on an everted intestine, jejunal absorption was slightly better than duodenal absorption, pointing to differences in the absorption transfer of AFB1 epithelial cells to blood circulation, making it dependent on intestinal tract section, and the animals growth and reproductive stages [56]. Previous studies on aflatoxin absorption toxicokinetics showed that aflatoxin’s uptake from the proximal intestine was very rapid. Following intraperitoneal application at a high dose of 20 mg/kg, AFB1 showed peak absorption in blood after 15 min, which was delayed to 30 min after oral ingestion in pregnant mice [57]. However, recirculation through bile was also very rapid, with an absorption rate of 5.0 µg/min and elimination of 3.0 µg/min, which could explain the slow transit progression observed in our study.

Mycotoxin binders are intended to protect animals against the mycotoxins’ toxic effects by adsorbing the toxin molecules. Bound toxins have reduced intestinal absorption, provided that the interaction between the binder and toxin is sufficiently strong to remain unaffected by the physiological changes encountered in the digestive tract. If the mitigation is efficient, mycotoxins are retained in the digesta and eventually removed from the body when excreted via feces [58]. In the present study, the animal diet contained YCW or HSCAS as an adsorbent and AFB1 as a toxin; the former was used at two different concentrations, namely 2.0 and 10 g/kg of feed, whereas the latter was administered at a unique dose of 10 g/kg of feed. We evaluated the effect of the two mycotoxin adsorbents in retaining AFB1 in the gastrointestinal tract. Our results revealed that the two adsorbents exhibited a highly significant propensity for maintaining higher toxin concentrations in the digestive compartment at both tested time points. This finding confirmed the ability of the adsorbents to limit the intestinal bioavailability of AFB1, leading to a decrease in the absorption of ^3^H-AFB1 through the intestine, which further confirmed the previously studied direct [25,46] and indirect mitigation effects observed in various animal species [31,32,44].

When mycotoxins are absorbed in livestock, the first systemic biological compartment where the toxin can be quantified is the blood [59], which becomes an interesting biological marker of AFB1 exposure in an animal organism. In our study, we were able to highlight both binders’ capacity in significantly decreasing the plasma concentration of AFB1 in rats subjected to dietary AFB1 exposure (Figure 5a). We can draw a parallel between this finding and recent findings obtained employing a bicameral Ussing chamber system in an ex vivo setup, in which a reduction in the transfer of AFB1 through the rat intestinal explants led to a decrease in the concentration of AFB1 in the serosal compartment following the use of both YCW and HSCAS [25]. Interestingly, when comparing the 5- and 10-h post-feeding timepoints of the present study, further accumulation of AFB1 could be observed over time, which was effectively prevented by both YCW and HSCAS. This finding also confirmed some of the results previously obtained in other animal species [48].

The liver is a vital organ when evaluating mycotoxicosis as it accumulates and metabolizes toxic compounds [60]. As such, it was expected that the radiolabeled aflatoxin would be detected at an appreciable concentration in the liver. Analysis of the accumulation of ^3^H-AFB1 in the liver yielded similar results to those observed in blood plasma (Figure 5b).

In our study, only a low proportion of the total radiolabeled AFB1 was found in the kidney. As expected, AFB1 only marginally accumulates in the kidney. Still, it is implicated in an indirect effect stemming from the activation of oxidative stress through modulation of L-proline levels [61] or an increase in urinary excretion of sodium and potassium and urinary gamma-glutamyl transferase and a decrease in glomerular filtration, reabsorption of glucose, or transport of *p*-aminohippurate [62].

As summarized in Figure 6, the two tested materials’ efficiency significantly decreased the absorption of ^3^H-AFB1 based on the recovered quantities from the intestinal digesta to systemic tissues in rats. The total amount of AFB1 in digesta and systemic samples, including plasma, liver, and kidney samples, showed a gradual decrease in the transfer via intestinal absorption of AFB1 with diet adsorbent inclusion. In contrast, an increase in the recovery of AFB1 was observed in the digesta in the presence of dietary absorbent, demonstrating the efficacy of the compartmentalization of AFB1 and the concomitant decrease in the bioavailability and ultimately sequestration of AFB1. The results observed were in line with those described by Firmin and coworkers [46], who analyzed radiolabeled AFB1 activity in feces, urine, and blood plasma following the oral administration of AFB1to rats fed diets containing or not containing YCW at two different doses. Results of that study showed that the proportion of radiolabeled AFB1 in feces increased significantly by 55% compared with that in the control group, with a concomitant decrease in urine, suggesting that AFB1 intestinal absorption was significantly reduced in rats fed a diet containing YCW. Interestingly, no dose-response relationship was observed in eliminating AFB1 in the test groups, potentially because there was a lack of response in the animal due to the low levels of AFB1 tested.

## 4. Conclusions

In this study, we evaluated the effects of an organic (YCW) and inorganic (HSCAS) adsorbent added to rats’ diets. We observed that at 5 and 10 h post-feeding, there was a significant effect on the pharmacokinetics of AFB1. The results accumulated throughout the study showed a consistent distribution of AFB1 in all digesta and tissue samples analyzed according to treatments, showing a significant decrease following treatment with YCW and HSCAS at 10 g/kg of feed and, to a lesser extent, following YCW treatment at 2.0 g/kg of feed. Taken together, the previous and current findings presented herein revealed the ability of YCW, to the same extent as that of HSCAS, efficiently adsorbing AFB1 in vivo, thus decreasing the toxin levels transferring across the digestive barrier to the systemic circulation of animals. Thus, contributing to the mitigation of the harmful effects of exposure to the AFB1 present in feed. The present study contributes to our understanding of the pharmacokinetics of AFB1 in an animal model, therefore, follow-up studies should focus on using more deterministic approaches such as employing adapted analytical methodologies (i.e., targeted metabolomics) to further elucidate the AFB1 metabolite profiles in the different animal compartments, and measure the animals inherent metabolic efficiency at detoxifying AFB1, in the presence or absence of a dietary mitigation aid.

## 5. Materials and Methods

### 5.1. In Vitro Main Study Assessing AFB1 Sequestration

A stock solution of 1.0 mg/mL AFB1 (Sigma Chemical Co., St. Louis, MO, USA) was prepared in acetonitrile. The accurate concentration of this stock solution was determined by spectrophotometry (λ_max_ = 362 nm; ε = 21,865). The adsorption efficacy of the two tested binders was determined in vitro with focus on the sub-parts per million levels of AFB1; five concentration points were evaluated: 0.05, 0.10, 0.25, 0.50, and 1.00 ng/mL. Three production batches of the YCW (Mycosorb^®^; Alltech Inc., Nicholasville, KY, USA) and one batch of HSCAS (bentonite T-150 containing >70% smectite (dioctahedral montmorillonite), Tolsa, Madrid, Spain) were tested for AFB1 at a concentration of 1 mg/mL.

Test concentrations for the main in vitro study were prepared by diluting the stock solution in 10 mM citrate buffer adjusted to pH 3.0 to match the physiological conditions of the proximal area of the digestive tract. Analysis was performed using a Waters Corp. (Milford, MA, USA) comprising an Acquity H-class ultra-performance liquid chromatograph fitted with a reverse phase C18 BEH analytical column (100 mm × 2.1 mm × 1.7 µm particle size) maintained at 30 °C and equipped with a fluorescent detector set up at excitation/emission wavelengths of 360/440 nm. The mobile phase consisted of ultrapure analytical grade water and acetonitrile both acidified with 0.1% formic acid (MS grade; Sigma-Aldrich, St-Louis, MO, USA) running in a gradient from 10% to 90% water over a period of 8 min and re-equilibration to the initial conditions for 1 min at a flow rate of 0.6 mL/min and injection volume of 5 µL. A 250-mL amber, silanized glass reaction bottle was used to allow precise weighing and correct mixing of the tested materials. After suspension of 100 mg of adsorbent in a volume of 100 mL (1 mg/mL) with each respective tested concentration of AFB1, samples were incubated for 90 min under orbital agitation (150 ppm) in an incubator maintained at 37 °C (I-series; New Brunswick™, Edison, NJ, USA).

### 5.2. In Vitro Kinetic of Adsorption

The responses were measured by integrating the chromatographic response area under the curve and calculating the corresponding concentration using a linear calibration curve forced through zero over the range of AFB1 tested concentrations. To measure the adsorption of the tested product, the amount of toxin bound was plotted against the amount of toxin initially added to the reaction media. The individual adsorption rate (ratio of toxin bound over the initial amount of toxin added) for every concentration point of toxin was calculated. The average adsorption rate for each product batch and the coefficient of variation were calculated. The kinetics of interaction were also evaluated using three individual equations previously considered suitable for sorbent evaluation [50], namely the Freundlich equation, Langmuir equation, and Hill’s model with the *n* sites equation, per previous studies [25,53], and establishing adsorption equilibrium, distribution constants, capacity, and intensity by evaluating the regression of the amount of toxin bound against the amount of free toxin at equilibrium in the reaction media using Datafit software (version 8.1.69, Oakdale Engineering, Oakdale, PA, USA)

### 5.3. Animals and Diets

Sprague–Dawley rats for the present study were obtained from Harlan Laboratories (Horst, The Netherlands). The animals were seven weeks old at the time of arrival and approximately nine weeks of age at the initiation of the study. The animal room was environmentally controlled, with a 12-h light/dark cycle with constant room temperature and humidity. Water was provided ad libitum, except during feeding time. The feeding period started in the mornings at 08.00–09.00 and in the evenings at 16.00–17.00. The feed base used was a Harlan special diet Teklad 2016 Global Rodent (Harlan—now Envigo, Madison, WI, USA—feed was produced in the Netherlands) containing by weight 16.4% proteins, 48.5% carbohydrate, 4.0% fat for a total energy density of 3.0 kcal/g. Crude fiber and neutral detergent fiber contributed to 3.3% and 15.2%, respectively. The Harlan Tecklad 2016 diet was amended with YCW and HSCAS material by the diet manufacturer at the tested inclusion rates.The target AFB1 dose was 0.2 µg per rat (25 µg/kg feed), including 0.63 µCi of [^3^H]-labeled AFB1 (Moravek Biochemicals Inc., Brea, CA, USA). The exact amount of labeled toxin was individually added to each 8-g dose of the experimental diet in a small amount of ethanol, which was evaporated prior to feeding.

### 5.4. Animal Pre-Experimental Study

A preliminary study was conducted to determine the absorption kinetics and distribution patterns of AFB1 in rats. Two rats were sacrificed at each of the following five-time points: 1, 2, 3, 5, and 7 h post-feeding, and ^3^H-AFB1 content in the jejunum, ileum, colon, plasma, liver, and kidney were measured quantitatively based on ^3^H radioactivity counting using a liquid scintillation counter.

### 5.5. Animal Principal Experimental Study

The trial was conducted in the research facility of Alimetrics, Ltd. (Espoo, Finland) in accordance with EU Directive 2010/63/EU. Following the standard operating procedures of Alimetrics Ltd., ethical approval or animal trial permit was not required because the substance under investigation is an approved feed ingredient in the EU, and the level of aflatoxin B1 included in the diets was below the EU regulatory levels. Animals were weighed and randomized into four groups of 16 animals and then identified with ear markings. The groups were divided in each cage into four separate parts using metal partitioning. The rats were conditioned for 9 days to eat 8-g diet portions immediately after the feed was provided. This was to ensure that all pellets containing the radiolabeled AFB1 were ingested within a short period of time. The rats were fasted between morning and evening feeding times. On day 10, after the administration of the radiolabeled feed, the cage was cleaned, the partitioning was removed, and water was provided ad libitum.

Study was performed on 16 rats per treatment. At 5 h, *n* = 9 rats for the 10 g/kg YCW treatment and *n* = 8 for the rest of the treatments were collected for analysis; at 10 h, the reminder rats (four rats were excluded due to morbidity/mortality issues before the start of the main experimental study period, *n* = 60) per treatments were collected for analysis, *n* = 6 in the control group and *n* = 7 in each of the adsorbent treated groups.

### 5.6. Sample Collection

At the 5 and 10 h sampling points, the rats were euthanized by CO_2_ inhalation, and blood was removed via cardiac puncture. Blood samples were drawn into heparinized syringes and transferred to heparinized test tubes for plasma separation via 5 min of centrifugation at 9000× *g*. The rats were then dissected, and their livers and kidneys were removed and rinsed with 0.9% NaCl. The gastrointestinal tract was separated into its constituent parts: the stomach, small intestine, cecum, and colon, and their contents were removed quantitatively for radioactivity counting. All samples were stored frozen at −20 °C until processed.

### 5.7. Radioactivity Determination

Frozen tissues were weighted, homogenized using pestle and mortar. The average weight of livers and kidneys collected was 11.2 and 2.3 g, respectively. An amount of 100 mg of homogenized tissue sample was placed into glass vials and dissolved with 2 mL of SOLVABLE™ aqueous-based tissue solubilizer (Perkin Elmer, Beaconsfield, UK) during 2 h at 60 °C. After cooling and solubilization, 300 μL of hydroxide peroxide 30% was added for color elimination and incubated during 30 min at 60 °C. After cooling, 15 mL of Ultima Gold™ scintillation liquid was added to each sample replicate for radioactive counting (Perkin Elmer, Waltham, MA, USA). The exact weight of each subsample and the total weight of the tissue sample collected were used in mass balance calculations.

Collected digesta were weighted and homogenized. An amount of 100 mg of digesta and 1 mL of plasma was processed via direct dample addition. A volume of 15 mL of Ultima Gold™ scintillation liquid was directly added to each sample replicate. For digesta compartments, sample size was dependent on the collection time point, and varied from 0.02 to 4.92 g. For the calculation of the total AFB1 in blood, an assumed blood volume of 20 mL was used.

The [^3^H] label was measured in duplicate using a scintillation counter (Microbeta 1450, Perkin Elmer, Waltham, MA, USA) using direct counting functionality of disintegrations per minute (DPM) accounting also for counting efficiency and any reduction in chemiluminescence.

### 5.8. Statistical Analysis

One-way Analysis of Variance (ANOVA) followed by a post-hoc test was performed for all measured parameters to test for differences between the treatments and to assess which treatments differed from each other. Dunnett’s post-hoc test was used to test the difference between the treatments and the negative control, and Tukey’s post-hoc test was used to compare all treatments pairwise. Multiple linear regression models were run for the doses of YCW, using the slope of each curve for each digesta and tissue samples and and based on 0, 20, and 100% of the YCW dose corresponding respectively to the control, 2 and 10 g/kg inclusion rate, to gain an understanding of how AFB1 levels in each tissue were affected by the respective doses used. All tests were performed in the SPSS statistical software package (IBM, version 22, Armonk, NY, USA) at a risk level of α = 0.05.

## Figures and Tables

**Table 1:** Measure of the adsorption rate (%) of aflatoxin B1 (AFB1) at each mycotoxin concentration point evaluate in three replicates and evaluation of the average individual adsorption rate (%) of three batches of yeast cell wall-based adsorbent (YCW) and one batch of hydrated sodium calcium aluminosilicate (HSCAS).

Tested Adsorbent:	YCW			HSCAS
AFB1 Concentration (μg/mL)	#496992	#496993	#496994	#50103379
1.00	85.58 ± 0.85	82.41 ± 0.85	81.53 ± 0.96	99.98 ± 0.03
0.50	87.75 ± 0.78	87.49 ± 2.24	85.27 ± 1.30	100.00
0.25	91.93 ± 0.38	88.61 ± 2.72	88.13 ± 1.94	100.00
0.10	93.88 ± 1.21	89.10 ± 0.26	88.93 ± 0.91	100.00
0.05	93.72 ± 0.45	92.72 ± 0.21	90.09 ± 1.68	100.00
Average (%)	90.57 ± 3.52 ^a^	88.07 ± 3.72 ^b,c^	86.79 ± 3.40 ^c^	100.00 ^d^
CV (%)	3.88	4.22	3.92	0.01
Average Total (%)	87.48 ± 3.73 ^a^			100.00 ^b^

One-factor ANOVA, Tukey’s post-hoc test (*p* < 0.05), and pairwise *t*-test. ^a,b,c,d^ Different superscript letters indicate significant differences between adsorbent treatments.

**Table 2:** Significance of the effect and percentage of changes observed for two mycotoxin adsorbents, yeast cell wall-based adsorbent (YCW) and hydrated sodium calcium aluminosilicate (HSCAS), on the distribution of ^3^H-labeled aflatoxin B1 (^3^H-AFB1) in the gastrointestinal digesta and in the tested organs and biological fluids of rats at 5 h post-feeding, as evaluated using three post-hoc statistical tests.

	ANOVA	Dunnett			MLR
Tissue		YCW 2 g/kg	YCW 10 g/kg	HSCAS 10 g/kg	YCW
Stomach (% DPM)		−16%	−25%	−8%	−21%
*Stomach (% recovery)*		−*31%*	−*31%*	−*9%*	−*27%*
Small intestine (% DPM)		+23%	+21%	+70%	+67% *
*Small intestine (% recovery)*		+*24%*	+*62%*	+*63%*	+58% **
Cecum (% DPM)	**	+26%	+70% **	+49% *	+66% ****
*Cecum (% recovery)*	**	+*20%*	+66% **	+49% *	+64% ****
Colon (% DPM)	*	+29%	+83%	+96% *	+78% *
*Colon (% recovery)*		+*27%*	+*78%*	+*123%*	+74% *
Total digesta (% DPM)		+8%	+32%	+35%	+31% *
*Total digesta (% recovery)*	****	+*6%*	+27% ***	+38% ****	+27% ****
Plasma (% DPM)	****	−15%	−50% ***	−67% ****	−49% ****
*Plasma (% recovery)*	****	−*16%*	−50% ***	−65% ****	−48% ***
Liver(% DPM)	****	−2%	−42% ****	−64% ****	−44% ****
*Liver (% recovery)*	****	−*5%*	−42% ***	−63% ****	−43% ***
Kidney (% DPM)	****	−8%	−37% ***	−54% ****	−37% ***
*Kidney (% recovery)*	***	−*11%*	−36% **	−52% ***	−35% ***
Total systemic (% DPM)	****	−9%	−46% ****	−66% ****	−46% ****
*Total systemic (% recovery)*	****	−*11%*	−46% ***	−64% ****	−45% ****

For each digesta or systemic tissue, the percent difference in tritiated label content coming from ^3^H-AFB1 (positive values indicating an increase, negative value a decrease) of each treatment compared to the control was calculated and statistically evaluated two ways: (1) In the first row based on the absolute level of tritium label (disintegration per minute, DPM) measured; and (2) in the second row, based on the recovery percentage of tritiated label (in percent) in each tissue. Dunnett’s test was performed against the values of rats fed unamended basic feed (negative control). Significant differences are indicated by asterisks as follows: * 0.01 ≤ *p* < 0.05; ** 0.001 ≤ *p* < 0.01; *** 0.0001 ≤ *p* < 0.001; **** *p* < 0.0001. Numbers in Dunnett’s and multiple linear regression (MLR) tests indicate the direction and magnitude of the effect. This study was performed initially on *n* = 64 rats, or 16 rats per treatment. At 5 h, *n* = 9 rats for the 10 g/kg YCW treatment and *n* = 8 for the rest of the treatments were collected for analysis. Integrality of each digestive compartiment and systemic tissue was collected for each rat.

**Table 3:** Significance of the effect and percentage of changes observed for two mycotoxin adsorbents, yeast cell wall-based adsorbent (YCW) and hydrated sodium calcium aluminosilicate (HSCAS), on the distribution of ^3^H-labeled aflatoxin B1 (^3^H-AFB1) in the gastrointestinal digesta and the tested organs and biological fluids of rats at 10 h post-feeding, as evaluated using three post-hoc statistical tests.

	ANOVA	Dunnett			MLR
Tissue		YCW 2 g/kg	YCW 10 g/kg	HSCAS 10 g/kg	YCW
Stomach (% DPM)		+363%	+844%	+506%	+788%
*Stomach (% recovery)*		+*347%*	+*824%*	+*413*	+*759%*
Small intestine (% DPM)		+13%	+160%	+49%	+167% **
*Small intestine (% recovery)*		+*6%*	+*129%*	+*45%*	+136% *
Cecum (% DPM)	**	+22%	+90% *	+86% **	+89% **
*Cecum (% recovery)*	***	+*15%*	+62% **	+80% ***	+61% **
Colon (% DPM)	*	+38%	+43%	+92% *	+32%
*Colon (% recovery)*	*	+*34%*	*+21%*	+83% **	+*11%*
Total digesta (% DPM)	****	+29%	+76% ***	+86% ***	+71% ***
*Total digesta (% recovery)*	****	+23% *	+50% ****	+79% ****	+46% ****
Plasma (% DPM)	****	−15%	−30% *	−64% ****	−25% *
*Plasma (% recovery)*	****	−22% **	−40% ****	−67% ****	−35% ***
Liver(% DPM)	****	−8%	−29% *	−58% ****	−28% **
*Liver (% recovery)*	****	−*14%*	−41% ***	−61% ****	−39% ***
Kidney (% DPM)	**	−12%	−15%	−49% **	−12%
*Kidney (% recovery)*	**	−*17%*	−29% *	−52% ***	−*25%*
Total systemic (% DPM)	****	−14%	−29% *	−61% ****	−26% **
*Total systemic (% recovery)*	****	−19% *	−41% ****	−64% ****	−37% ****

For each digesta or systemic tissue, the percent difference in tritiated label content coming from 3H-AFB1 (positive values indicating an increase, negative value a decrease) of each treatment compared to the control was calculated and statistically evaluated two-ways: 1-In the first row based on the absolute level of tritium label (disintegration per minute, DPM) measured; 2-In the second row, based on the recovery percentage of tritiated label (in percent) in each tissue. Dunnett’s test was performed against the values of rats fed unamended basic feed (negative control). Significant differences are indicated by asterisks as follows: * 0.01 ≤ *p* < 0.05; ** 0.001 ≤ *p* < 0.01; *** 0.0001 ≤ *p* < 0.001; **** *p* < 0.0001. Numbers in Dunnett’s and multiple linear regression (MLR) tests indicate the direction and magnitude of the effect. This study was performed on *n* = 64 rats, or 16 rats per treatment. At 10 h, the reminder rats after 5 h collection (four rats were excluded due to morbidity/mortality issues before the start of the main experimental study period) per treatments were collected for analysis, *n* = 6 in the control group and *n* = 7 in each of the adsorbent treated groups. Integrality of each digestive compartiment and systemic tissue was collected for each rat.

## Data Availability

Data will be provided upon request.
